# The corrosion resistance, cytotoxicity, and antibacterial properties of lysozyme coatings on orthodontic composite arch wires

**DOI:** 10.1039/d0ra02988b

**Published:** 2020-05-12

**Authors:** Longwen He, Ye Cui, Chao Zhang

**Affiliations:** Orthodontic Department, Stomatological Hospital, Southern Medical University Guangzhou 510280 China 2645491781@qq.com +86 18565578907

## Abstract

*Objective*: The corrosion resistance of new orthodontic composite arch wires (CAWs), which have excellent mechanical properties in a simulated oral environment, must be improved. This study explored the susceptibility to corrosion, *in vitro* cytotoxicity, and antibacterial properties of lysozyme-coated CAWs. *Methods*: Lysozyme coating of laser-welded CAW surfaces was prepared by liquid phase deposition. Four groups of CAW specimens were prepared: uncoated CAWs and CAWs coated with 20, 40, and 60 g L^−1^ lysozyme. The surface morphology of the lysozyme coatings was characterized by atomic force microscopy. The samples were immersed in artificial saliva (AS) for 2 weeks, and corrosion morphology was then observed by scanning electron microscopy. Corrosion behavior was characterized according to weight loss and electrochemical properties. The cytotoxicity and antibacterial properties of lysozyme-coated CAWs were assessed by cell counting kit-8 assay and a live/dead bacterial test, respectively. *Results*: Surfaces in the three lysozyme coating groups exhibited film-like deposition, the thickness of which increased with the lysozyme concentration. Surface pitting and copper ion precipitation decreased with increasing lysozyme concentration in coatings. The corrosion tendency declined as the corrosion and pitting potentials decreased. The corrosion morphology and electrochemical parameters together indicated that lysozyme coatings increased corrosion resistance. The coatings also reduced cytotoxicity to L-929 cells and increased anti-*Staphylococcus aureus* ability. *Conclusions*: Lysozyme coating of CAW surfaces by liquid phase deposition improved the corrosion resistance of CAWs. The protective coatings improved biocompatibility and endowed the CAW surfaces with certain degrees of anti-*Staphylococcus aureus* activity. Different lysozyme concentrations had different protective effects, with 40 g L^−1^ maybe being the ideal lysozyme concentration for CAW coatings.

## Abstract 2

The corrosion resistance of new orthodontic composite arch wires (CAWs), which have excellent mechanical properties in a simulated oral environment, must be improved.

## Graphical abstract

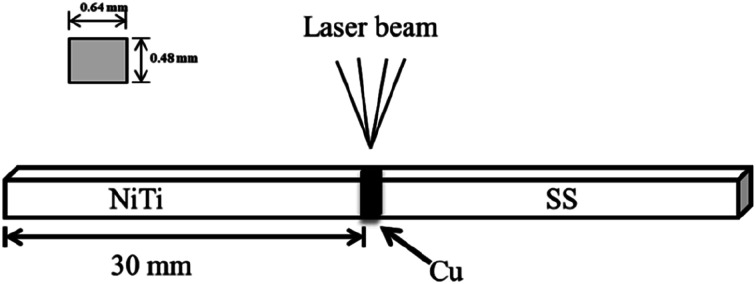

## 1. Introduction

Orthodontic composite arch wires (CAWs) are made of nitinol alloy (NiTi) and stainless steel (SS), and are produced by laser welding with copper (Cu) serving as the interlayer.^1^ They have excellent mechanical properties for orthodontic application; the NiTi segment exerts the appropriate orthodontic force on dislocated teeth, and the SS segment provides anchorage for anchoring teeth.^2^ However, the Cu interlayer of CAWs made of heterogeneous alloys by welding may be damaged in the corrosive oral environment.^3^ Severe corrosion leads to CAW fracture and damage of the adjacent mucosa; mild corrosion can also cause the rupture of the passivation film on the surface and increased friction between the CAW and bracket, leading to the resistance of tooth movement.^4^ Ion release accompanied by corrosion can induce gingivitis, allergic reactions, and other side effects.^5–7^ Moreover, the complex arch wire and brackets are difficult to clean, and accumulated dental plaque can cause enamel demineralization, dental caries, and periodontal disease. Without effective intervention, demineralization occurs in 50–60% of orthodontic patients.^8^ Thus, a surface modification strategy with good biosafety for orthodontic appliances is needed to prevent and control plaque accumulation, reduce bacterial adhesion and corrosion, and improve orthodontic accessory structures.

Lysozyme is an important antibacterial component that is extensively present in mammalian serum, liver, secretions (*i.e.*, saliva, tears, urine, and milk), and immune cells, and on mucosal surfaces.^9,10^ It has an antimicrobial effect *via* selective decomposition of the cell walls of microorganisms, with no destruction of other tissues.^11,12^ As the cornerstone of innate immunity, lysozyme is involved in the homeostasis of the oral environment, the disruption of which is related to the occurrence and development of dental caries,^13^ oral mucosal disease,^14,15^ and periodontal disease.^16^

Certain protein components can improve the corrosion resistance of alloys,^17,18^ possibly through the coupling of metal oxidation with the reduction of oxygen to form protein–metal complexes.^19^ Lysozyme, as a beneficial protein component in the oral environment, may show promise for alloy surface modification. In this study, CAWs coated with different concentrations of lysozyme were prepared by liquid phase deposition (LPD), and their corrosion morphologies and electrochemical properties were examined to explore CAW corrosion behavior. The antibacterial performance and *in vitro* cytotoxicity of the lysozyme-coated CAWs were also evaluated.

## 2. Materials and methods

### 2.1 CAW preparation

For the experiment, NiTi shape memory alloy wires (55.2–56.2 wt% Ni; Grikin Co., Beijing, China) and austenitic SS wires (Fe–19Cr–10Ni; Kangqiao Dental Instruments Factory, Shanghai, China) were used as the base materials for CAWs. Wires of both types with rectangular shape had cross-sectional dimensions of 0.48 × 0.64 mm and lengths of 30 mm. They were polished with waterproof silicon carbide abrasive papers, and then cleaned ultrasonically with acetone followed by distilled water, and finally dried. Then, as described previously,^3^ the NiTi and SS wires were welded with a pure Cu interlayer using a 1064 nm Q-switched neodymium-doped yttrium aluminum garnet laser (JHM-1GY-300B; Chutian Laser Group Co., Ltd., Wuhan, China). The surface morphology and microstructure of the CAW samples were observed under a Hitachi scanning electron microscope (S-3400N; Hitachi Koki Scientific Instruments Co., Ltd., Tokyo, Japan). Moreover, in order to determine whether the interlayer exhibit a heterogeneous composition, energy-dispersive X-ray spectroscopy (EDS) with an EMAX controller (Horiba, Tokyo, Japan) was selected to characterize the interlayer, *i.e.* the welding area, of the CAW sample.

### 2.2 Coating formation and characteristics

Artificial saliva (AS, ISO/TR10271) was obtained from Leagene Biotechnology (Beijing, China), and its pH was adjusted to 4 with lactic acid. To functionalize the CAW surfaces with lysozyme (Solarbio Life Sciences, Beijing, China) coating, the alloy substrates were immersed in AS solutions containing different concentrations (20, 40, and 60 g L^−1^) of lysozyme with continuous stirring for 48 h at 37 °C. After coating protein deposition, the samples were taken out of the solutions, washed gently with pure water to eliminate remaining impurities, and dried at 50 °C for 24 h. Their weights were then recorded using an electronic analytical balance (EX-H; Zanwei Co., Ltd., Beijing, China). CAWs with no lysozyme coating served as controls. The topographical characteristics of randomly selected coated and uncoated CAWs and were examined in random fields under an atomic force microscope (AFM; Agilent Inc., Pittsfield, MA, USA).

### 2.3 Immersion tests and scanning electron microscopic observation

The CAW samples were immersed in AS for 2 weeks at 37 °C. After removal from the AS, the leach solutions were collected for ion release analysis and subsequent biocompatibility assay; the samples were subjected to microbiological testing. Five replicates per group were prepared. The release of Cu ions in the leach solutions was analyzed by inductively coupled plasma optical emission spectrometry (iCAP™ 7000; Thermo Fisher Scientific, Shanghai, China). The CAWs were weighed again, and the differences from the pre-immersion weights were taken as the weight losses due to corrosion. The surface morphology of the CAWs was re-examined by scanning electron microscopy (SEM) to identify corrosion.

### 2.4 Electrochemical testing

Electrochemical tests were performed to evaluate the corrosion behavior of the coated CAWs. The surfaces of the CAW samples were sealed with epoxy resin, leaving only an area of 20 × 0.64 mm^2^ exposed, connected with copper wire. Each sample served as the working electrode, platinum served as the auxiliary electrode, and a saturated calomel electrode served as the reference electrode connected to the electrochemical workstation (CHI 920C; CH Instruments Inc., Shanghai, China). AS was used as the electrolyte for detection. For all potentiodynamic polarization detections, potentials were measured from −1000 mV to 1000 mV at a scan rate of 5 mV s^−1^. The measurement was repeated six or more times, with a new corrosion solution and sample used each time until the basic trend of the polarization curve was consistent. The polarization curves were used to evaluate the corrosion potential (*E*_corr_), pitting potential (*E*_pit_), and corrosion current density (*I*_corr_).

### 2.5 *In vitro* biocompatibility assay

The biocompatibility of lysozyme-coated CAWs was tested using the L-929 murine embryonic fibroblast cell line (Zhongqiao Xinzhou Co., Ltd., Shanghai, China). The cells were cultured in Dulbecco's Modified Eagle's Medium (DMEM; Thermo Fisher Scientific) containing 10% fetal bovine serum and 1% penicillin–streptomycin (100 U mL^−1^ penicillin and 100 μg mL^−1^ streptomycin). They were then inoculated in an incubator at 37 °C with 5% CO_2_ and 95% humidity.

Cytotoxicity was examined using a cell counting kit-8 (CCK-8; Dojindo Laboratories, Shanghai, China). L-929 cells (9 × 10^3^ per well) were seeded in 96-well plates (Corning Inc., Corning, NY, USA) for 24 h at 37 °C with 5% CO_2_ and 95% humidity. Extraction solutions were prepared using DMEM as the extraction medium, with a surface area ratio of 0.2 g mL^−1^. Then, the extraction solutions for each group were then added, followed by 24 h co-culture. For the positive control, the cells were exposed for the same amount of time to dimethyl sulfoxide (Rhawn, Beijing, China). DMEM was used for the negative control. Then, CCK-8 reagent was added to each well, followed by incubation at 37 °C for 1 h. Cell proliferation activity was evaluated according to the supplier's protocol. In this assay, L-929 cell viability reflects not only the cytotoxicity of the lysozyme coatings, but also the extent of corrosion. Absorbance was detected at 450 nm using an ultra-microplate reader (Reagen, Beijing, China).

### 2.6 Antibacterial property detection

Gram-positive *Staphylococcus aureus* (ATCC 25923) was used to determine the antibacterial activity of the coated CAWs. The test strain was incubated with a cells density of (∼10^8^ cells per mL) for 1 h in a static incubator at 37 °C with 5% CO_2_ and 95% humidity. Bacterial suspension was added to co-culture with CAW samples for 24 h. Thereafter, samples with biofilms were cleaned with cysteine peptone water to remove non-adhering bacteria. Each sample was stained with a mixture of dimethyl sulfoxide (1×) and ethidium homodimer III (2×) using a live/dead bacterial staining kit (Thermo Fisher Scientific) according to the supplier's protocol. The biofilms were examined by confocal laser scanning microscopy (5 EXCITER; Analytik Jena AG Inc., Jena, Germany). All experimental data were derived from three independent experiments.

### 2.7 Statistical analysis

The data are expressed as means ± standard deviations from three independent experiments. All analyses were carried out using GraphPad Prism (GraphPad Software, Inc., La Jolla, CA, USA) using the unpaired Student's *t* test and one-way analysis of variance (ANOVA). The data were considered to be statistically significant when the probability (*p*) < 0.05.

## 3. Results

### 3.1 CAW characteristics

**Fig. 1:**
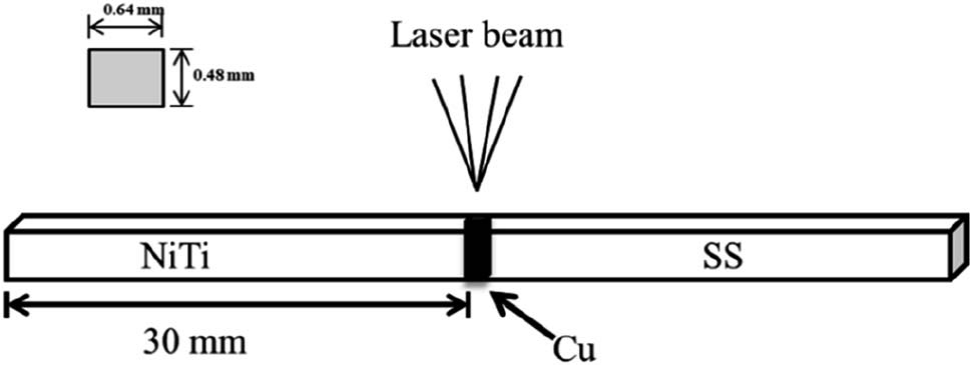
Schematic diagram of a composite arch wire produced by laser welding. NiTi, nitinol alloy; SS, stainless steel; Cu, copper.

**Fig. 2:**
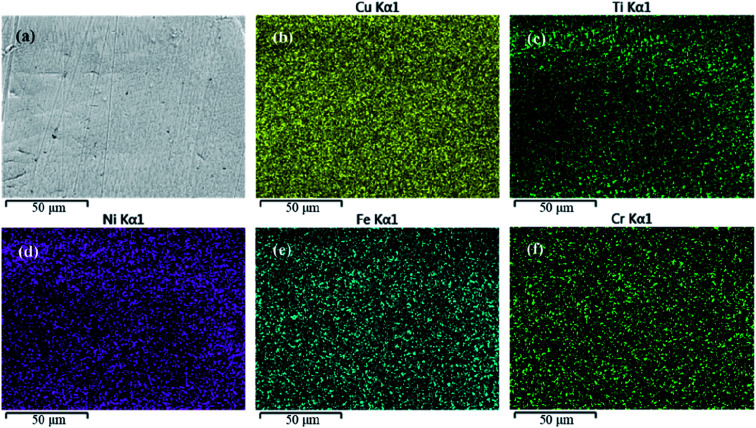
(a) Representative scanning electron micrograph of the composite arch wire interlayer and (b–f) energy-dispersive X-ray spectroscopic images of individual components. Cu, copper; Ti, titanium; Ni, nickel; Fe, iron; Cr, chromium.

**Fig. 3:**
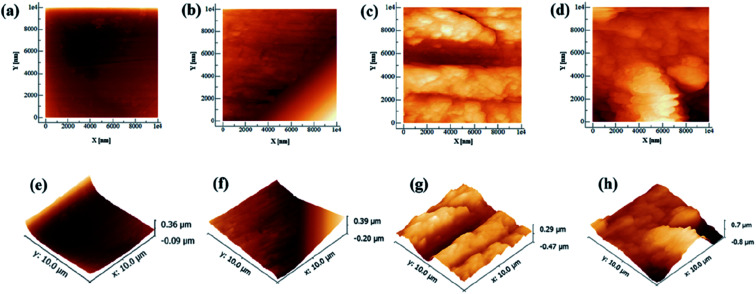
Representative two- and three-dimensional atomic force micrographs of coated composite arch wires (CAWs). (a and e) Uncoated CAW. (b and f) CAW coated with 20 g L^−1^ lysozyme. (c and g) CAW coated with 40 g L^−1^ lysozyme. (d and h) CAW coated with 60 g L^−1^ lysozyme.

A schematic diagram of a CAW is provided in Fig. 1. EDS showed that the CAW interlayer was composed of Cu, Ti, Ni, iron, and chromium, distributed uniformly due to laser welding (Fig. 2). The three lysozyme coating groups exhibited film-like surface deposition (Fig. 3). These deposits were thin in the 20 g L^−1^ lysozyme group; more obvious, with dispersive mountain-like morphology, in the 40 g L^−1^ lysozyme group; and thickest, with no gap between protuberances, in the 60 g L^−1^ lysozyme group.

### 3.2 Surface morphology of coated CAWs after immersion in AS

**Fig. 4:**
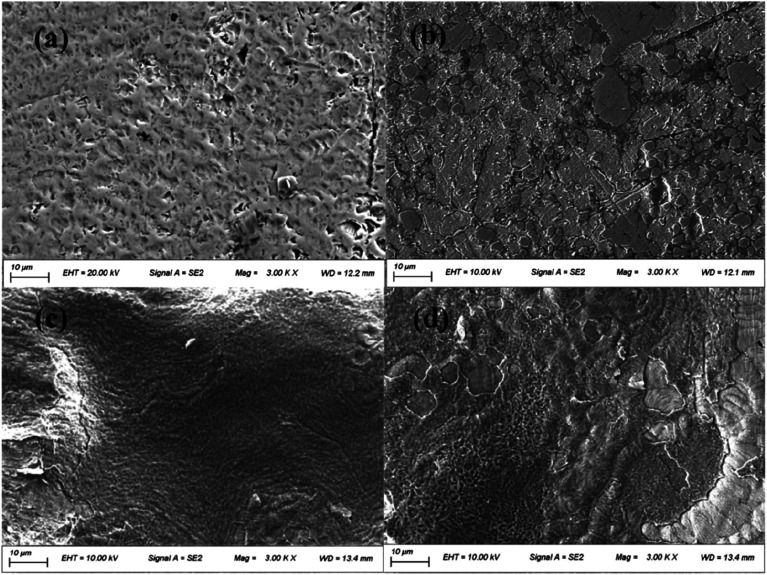
Scanning electron micrographs of composite arch wire (CAW) interlayers after immersion in artificial saliva for 2 weeks. (a) Uncoated CAW and (b–d) CAWs coated with 20, 40, and 60 g L^−1^ lysozyme, respectively. EHT, extra high tension. SE, secondary electrons. Mag, magnification. WD, working distance.

After soaking in AS for 2 weeks, the surfaces of the uncoated CAWs were rough, with abundant pitting (Fig. 4a). Parts of the surfaces coated with 20 g L^−1^ lysozyme were covered by small pieces of membrane structures, with small superficial corrosion pits visible in the uncovered areas (Fig. 4b). CAWs coated with 40 g L^−1^ lysozyme showed relatively smooth surfaces with no obvious pitting (Fig. 4c). The superficial layers of surfaces coated with 60 g L^−1^ lysozyme were disrupted at some sites, and the surface morphology was rougher than that in the 40 g L^−1^ group, likely reflecting peeling (Fig. 4d); these samples showed no obvious corrosion-related pitting.

### 3.3 Release of Cu ions and weight loss

**Fig. 5:**
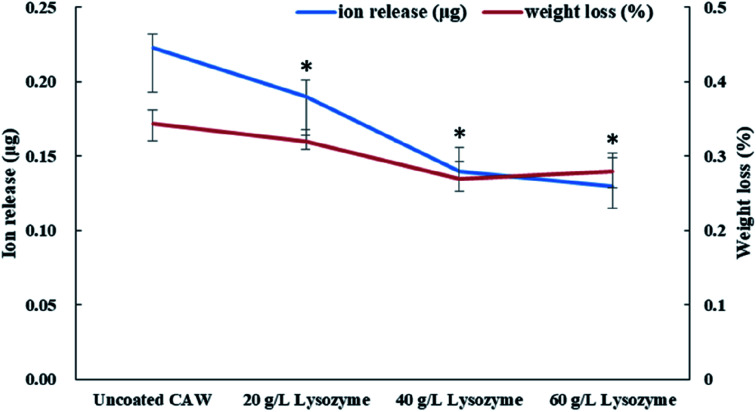
Copper ion release and weight loss of composite arch wires (CAWs) coated with different concentrations of lysozyme after 2 weeks of immersion in artificial saliva. All data expressed as mean ± standard deviations in three independent experiment results. *p < 0.05 *vs.* control group.

Coated CAWs exhibited less Cu ion release and weight loss than did uncoated CAWs, and Cu ion release decreased with increasing lysozyme concentration (Fig. 5). The difference in weight loss among lysozyme groups was slight, as the deposition of lysozyme molecules compensated the lost weight.

### 3.4 Electrochemical properties

**Fig. 6:**
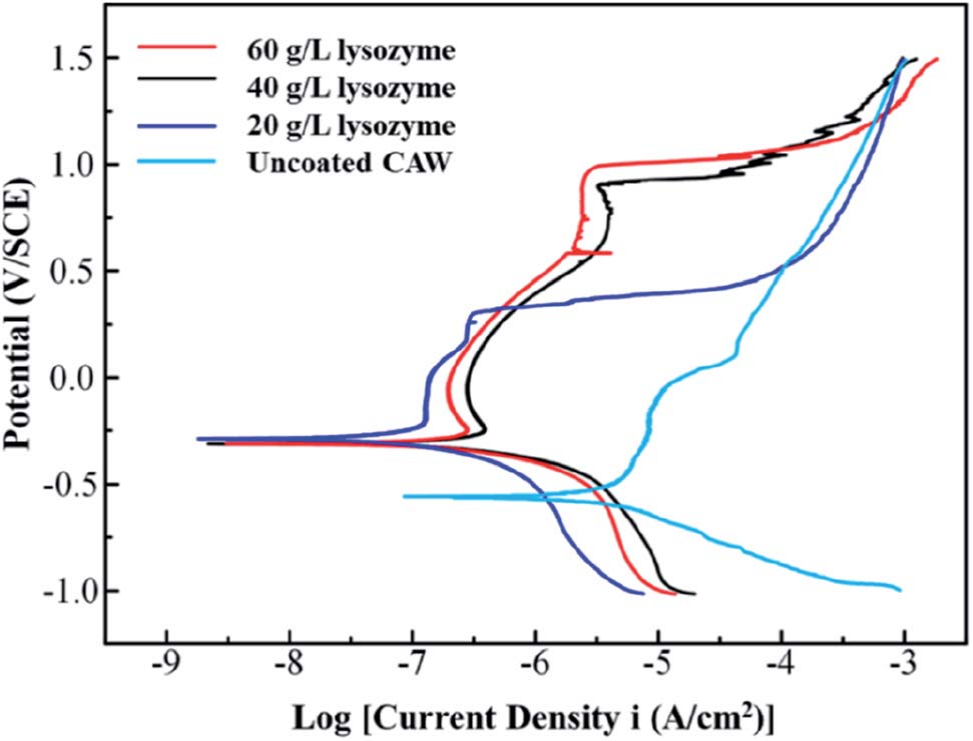
Potentiodynamic polarization curves of composite arch wires (CAWs) with and without lysozyme coating. SCE, saturated calomel electrode.

The polarization curves from which the electrochemical parameters of the CAWs were derived are shown in Fig. 6. *E*_corr_ and *E*_pit_ values were higher for coated than for uncoated CAWs (Table 1, Fig. 6). *E*_pit_, but not *E*_corr_, values increased significantly with the lysozyme concentration. *I*_corr_ values were also higher for coated than for uncoated CAWs.

**Table 1:** *E*_corr_, *E*_pit_, and *I*_corr_ values calculated from potentiodynamic polarization curves^a^

Sample	*E*_corr_ (mV/SCE)	*E*_pit_ (mV/SCE)	*I*_corr_ (μA cm^−2^)
Uncoated CAW	−574 ± 23	111 ± 14	1.10 ± 0.13
20 g L^−1^ lysozyme	−291 ± 11	295 ± 16	2.09 ± 0.10
40 g L^−1^ lysozyme	−299 ± 09	867 ± 14	7.09 ± 0.29
60 g L^−1^ lysozyme	−301 ± 12	1014 ± 7	6.43 ± 0.27

a Data are presented as means ± standard deviations. *E*_corr_, corrosion potential; *E*_pit_, pitting potential; *I*_corr_, corrosion current density; SCE, saturated calomel electrode; CAW, composite arch wire.

### 3.5 *In vitro* cytotoxicity and antibacterial activity of CAWs

**Fig. 7:**
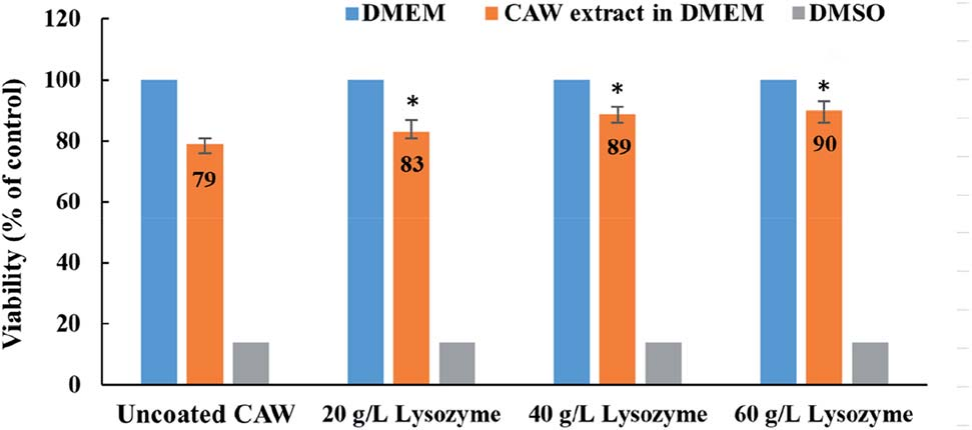
Cell viability for composite arch wires (CAWs) coated with different concentrations of lysozyme. DMEM, Dulbecco's Modified Eagle's Medium; DMSO, dimethyl sulfoxide. All data expressed as mean ± standard deviations in three independent experiment results. *p < 0.05 *vs.* control group.

**Fig. 8:**
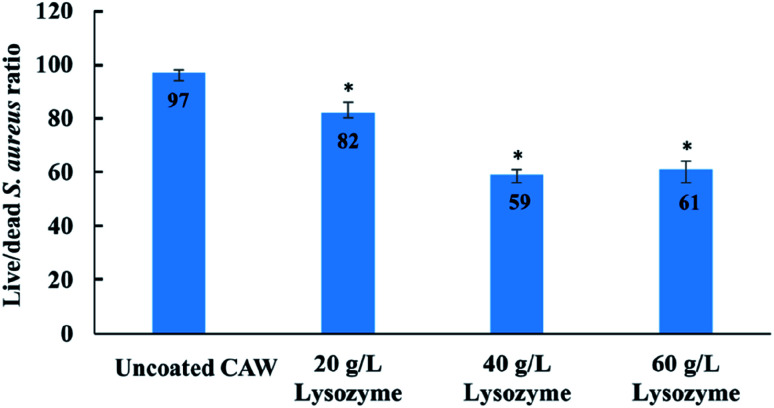
Live/dead bacteria staining results for composite arch wires (CAWs) coated with different concentrations of lysozyme. All data expressed as mean ± standard deviations in three independent experiment results. *p < 0.05 *vs.* control group.

Cell viability was greater for coated than for uncoated CAWs, and increased with the lysozyme concentration (Fig. 7). The uncoated CAWs showed very little antibacterial activity, with live/dead bacteria ratios >95% (Fig. 8). This ratio was lower for coated CAWs, with the lowest value of <60% observed for CAWs coated with 40 g L^−1^ lysozyme.

## 4. Discussion

The oral cavity is a humid and highly corrosive complex physiological environment with a large number of microorganisms.^20^ When orthodontic appliances are used in the long-term treatment, they are faced with not only complex electrochemical environment but bacterial adhesion and the metabolism of acid production, leading to the weakening of metal corrosion resistance. We use a simple methods to modified the surface of CAW, and the corrosion resistance, cytotoxicity, and antibacterial properties was evaluated.

In recent years, extensive studies have examined a variety of surface modification methods to improve corrosion resistance and biocompatibility,^21,22^ such as chemical vapor deposition,^23^ physical vapor deposition,^24^ chemical and electrochemical techniques,^25^ and thermal^26^ and plasma^27^ spraying. LPD technology, developed from wet chemical processes, is used widely in the preparation of functional coatings from aqueous solutions.^28^ Benzaldehyde and anisaldehyde have been used to chemically modify lysozyme for the extracorporeal treatment of sepsis,^29^ but the introduction of toxic chemical compounds could be harmful. Some methods of lysozyme modification, such as thermochemical modification, could cause oligomerization of the enzyme and express greater antibacterial activity.^30^ Thus, the deposition of lysozyme together with calcium and phosphate in AS *via* LPD at high temperature produces surfaces with greater antibacterial activity. Lysozyme is rich in basic amino acids, and stable in acidic media. It is heat resistant, maintaining 87% activity for 15 min at 96 °C and a pH of 3. In the present study, AFM confirmed that the coating of CAWs with lysozyme by LPD was successful, and conferred considerable antibacterial capacity.

In this study, CAWs coated with 20 g L^−1^ lysozyme showed fewer corrosion pits and lesser corrosion depths relative to uncoated CAWs, with significantly increased *E*_pit_ and less Cu ion precipitation, indicating that corrosion was slowed compared with the control. Corrosion resistance was further enhanced in CAWs coated with 40 and 60 g L^−1^ lysozyme. The lysozyme coating blocks charge-transfer reactions between the electrolyte and CAW substrate, thereby preventing substrate corrosion.^31^

The deposition of effective coating components on the substrate increased with the lysozyme concentration. During coating formation by LPD, calcium and phosphate ions are adsorbed, nucleated together with lysozyme molecules, and finally deposited on the CAW interface. With the continuous accumulation of particles, the extent of coating gradually expands and the thickness gradually increases; in this study, AFM and SEM confirmed the occurrence of this process, and its correlation with the lysozyme concentration. The 20 g L^−1^ lysozyme coating did not adequately prevent corrosion; the 40 g L^−1^ coating showed increased anti-corrosion ability. The electrochemical properties and Cu ion release of the 60 g L^−1^ lysozyme coating were very similar to those of the 40 g L^−1^ lysozyme coating, with the former showing a rougher surface topography. During film drying, cracks appear due to dehydration; the internal stress is greater for thicker films, promoting the generation of wider and deeper cracks, which results in increased surface roughness and more susceptibility to pitting corrosion, as observed in the 60 g L^−1^ lysozyme group in this study. Stable coatings are supposed to be similar to the passive films formed early in some corrosive solutions that we examined previously.^2,17^ Thus, 40 g L^−1^ may be the best lysozyme concentration for CAW coating by LPD, which may be applied in future clinical studies.

The slight and dose-dependent increase in L-929 cell viability observed for coated CAWs in this study confirmed the biocompatibility of the coatings. Cell damage was reduced due to the greater corrosion resistance and lesser metal ion release of the coated CAWs.

*S. aureus* is considered to be a major human pathogen, and it has a great importance also in dentistry. Together with *Staphylococcus epidermidis*, is considered to be the putative cause of 80–90% of osteomyelitis of the jaw.^32^*S. aureus* is also an important pathogenic factor of oral mucosal infection. The results of live/dead bacteria staining showed that the coated CAWs had better antibacterial capacity than did the uncoated CAWs, although it did not go up as expected in the 60 g L^−1^ lysozyme group relative to the 40 g L^−1^ lysozyme group. One possible reason is that when the thickness of the coating increases over a certain value, it means that the surface roughness may be further increased and the bacterial adhesion may be enhanced, while the antibacterial effect will be restricted.^33^ These results were also reported in other previous study, which showed that it was not always the higher the concentration of lysozyme, the better the bactericidal effect was.^34^

It is showed that the antibacterial effect of 20 g L^−1^ lysozyme is much weaker than that of 40 g L^−1^, while the antibacterial effect of 60 g L^−1^ was similar as 40 g L^−1^ rather than went up. This means that there was a range of optimum concentrations of the coating liquor, which is between 40 g L^−1^ and 60 g L^−1^. It is speculated that the increased bacterial adhesion was the reason, due to the larger surface roughness of 60 g L^−1^ group in the results of SEM and AFM.^35^ Furthermore, considering that the friction between the arch wire and the bracket may be affected by the roughness of coating, 40 g L^−1^ lysozyme could be the ideal concentration in the future clinical studies.

## 5. Conclusions

Lysozyme coatings for CAWs can be prepared by LPD. In this study, these coatings improved the AS corrosion resistance of the fragile CAW interlayer, reduced the precipitation of toxic Cu ions, improved biocompatibility, and endowed CAW surfaces with certain degrees of anti-*S. aureus* activity. Different concentrations of lysozyme had different effects on corrosion resistance, antibacterial properties, and cytotoxicity, with 40 g L^−1^ may be the ideal lysozyme concentration for CAW coatings.

## Conflicts of interest

The authors declare no conflicts of interest in relation to this paper.

## Supplementary Material
